# Supplementary material for: Unveiling the Bioactive Potential of the Invasive Jellyfish Phyllorhiza punctata Through Integrative Transcriptomic and Proteomic Analyses
Source: Biomolecules. 2025 Aug 4;15(8):1121. doi: 10.3390/biom15081121 (PMC12383608; doi:10.3390/biom15081121)
Supplement: Supplementary file 1 [file biomolecules-15-01121-s001.zip › Suplementary Material/FileS3/PEPFOLD4/XP_065059012_5408/clusters.html]

 PEP-FOLD clusters


Download archive of all models  here   

|  |  |  |
| --- | --- | --- |
| # | file | sOPEP |
| # cluster 1 - Size: 33 |  |  |
|  | XP\_065059012\_5408-model1 | -8.41553 |
|  | XP\_065059012\_5408-model1.1 | -8.41553 |
|  | XP\_065059012\_5408-model1.2 | -8.41553 |
|  | XP\_065059012\_5408-model1.3 | -8.41553 |
|  | XP\_065059012\_5408-model1.4 | -8.41553 |
|  | XP\_065059012\_5408-model1.5 | -8.41553 |
|  | XP\_065059012\_5408-model1.6 | -8.41553 |
|  | XP\_065059012\_5408-model1.7 | -8.41553 |
|  | XP\_065059012\_5408-model1.8 | -8.41553 |
|  | XP\_065059012\_5408-model1.9 | -8.41553 |
|  | XP\_065059012\_5408-model1.10 | -8.41553 |
|  | XP\_065059012\_5408-model1.11 | -8.41553 |
|  | XP\_065059012\_5408-model1.12 | -8.41553 |
|  | XP\_065059012\_5408-model1.13 | -8.41553 |
|  | XP\_065059012\_5408-model1.14 | -8.41553 |
|  | XP\_065059012\_5408-model1.15 | -8.41553 |
|  | XP\_065059012\_5408-model1.16 | -8.41553 |
|  | XP\_065059012\_5408-model1.17 | -8.41553 |
|  | XP\_065059012\_5408-model1.18 | -8.41553 |
|  | XP\_065059012\_5408-model1.19 | -8.41553 |
|  | XP\_065059012\_5408-model1.20 | -8.41553 |
|  | XP\_065059012\_5408-model1.21 | -8.41553 |
|  | XP\_065059012\_5408-model1.22 | -8.41553 |
|  | XP\_065059012\_5408-model1.23 | -8.41553 |
|  | XP\_065059012\_5408-model1.24 | -8.41553 |
|  | XP\_065059012\_5408-model1.25 | -8.41553 |
|  | XP\_065059012\_5408-model1.26 | -8.41553 |
|  | XP\_065059012\_5408-model1.27 | -8.41553 |
|  | XP\_065059012\_5408-model1.28 | -8.41553 |
|  | XP\_065059012\_5408-model1.29 | -8.41553 |
|  | XP\_065059012\_5408-model1.30 | -8.41553 |
|  | XP\_065059012\_5408-model1.31 | -8.41553 |
|  | XP\_065059012\_5408-model1.32 | -8.30295 |
| # cluster 2 - Size: 14 |  |  |
|  | XP\_065059012\_5408-model2 | -8.37488 |
|  | XP\_065059012\_5408-model2.1 | -8.37488 |
|  | XP\_065059012\_5408-model2.2 | -8.37488 |
|  | XP\_065059012\_5408-model2.3 | -8.37488 |
|  | XP\_065059012\_5408-model2.4 | -8.37488 |
|  | XP\_065059012\_5408-model2.5 | -8.37488 |
|  | XP\_065059012\_5408-model2.6 | -8.37488 |
|  | XP\_065059012\_5408-model2.7 | -8.37488 |
|  | XP\_065059012\_5408-model2.8 | -8.37488 |
|  | XP\_065059012\_5408-model2.9 | -8.37488 |
|  | XP\_065059012\_5408-model2.10 | -8.37488 |
|  | XP\_065059012\_5408-model2.11 | -8.32616 |
|  | XP\_065059012\_5408-model2.12 | -8.32616 |
|  | XP\_065059012\_5408-model2.13 | -8.32616 |
| # cluster 3 - Size: 39 |  |  |
|  | XP\_065059012\_5408-model3 | -8.36348 |
|  | XP\_065059012\_5408-model3.1 | -8.36348 |
|  | XP\_065059012\_5408-model3.2 | -8.36348 |
|  | XP\_065059012\_5408-model3.3 | -8.36348 |
|  | XP\_065059012\_5408-model3.4 | -8.36348 |
|  | XP\_065059012\_5408-model3.5 | -8.36348 |
|  | XP\_065059012\_5408-model3.6 | -8.36348 |
|  | XP\_065059012\_5408-model3.7 | -8.36348 |
|  | XP\_065059012\_5408-model3.8 | -8.36348 |
|  | XP\_065059012\_5408-model3.9 | -8.36348 |
|  | XP\_065059012\_5408-model3.10 | -8.36348 |
|  | XP\_065059012\_5408-model3.11 | -8.36348 |
|  | XP\_065059012\_5408-model3.12 | -8.36348 |
|  | XP\_065059012\_5408-model3.13 | -8.36348 |
|  | XP\_065059012\_5408-model3.14 | -8.36348 |
|  | XP\_065059012\_5408-model3.15 | -8.36348 |
|  | XP\_065059012\_5408-model3.16 | -8.36348 |
|  | XP\_065059012\_5408-model3.17 | -8.36348 |
|  | XP\_065059012\_5408-model3.18 | -8.36348 |
|  | XP\_065059012\_5408-model3.19 | -8.36348 |
|  | XP\_065059012\_5408-model3.20 | -8.36348 |
|  | XP\_065059012\_5408-model3.21 | -8.36348 |
|  | XP\_065059012\_5408-model3.22 | -8.36348 |
|  | XP\_065059012\_5408-model3.23 | -8.36348 |
|  | XP\_065059012\_5408-model3.24 | -8.36348 |
|  | XP\_065059012\_5408-model3.25 | -8.34306 |
|  | XP\_065059012\_5408-model3.26 | -8.34306 |
|  | XP\_065059012\_5408-model3.27 | -8.34306 |
|  | XP\_065059012\_5408-model3.28 | -8.34306 |
|  | XP\_065059012\_5408-model3.29 | -8.34306 |
|  | XP\_065059012\_5408-model3.30 | -8.34306 |
|  | XP\_065059012\_5408-model3.31 | -8.34306 |
|  | XP\_065059012\_5408-model3.32 | -8.34306 |
|  | XP\_065059012\_5408-model3.33 | -8.34306 |
|  | XP\_065059012\_5408-model3.34 | -8.34306 |
|  | XP\_065059012\_5408-model3.35 | -8.34306 |
|  | XP\_065059012\_5408-model3.36 | -8.34306 |
|  | XP\_065059012\_5408-model3.37 | -8.34306 |
|  | XP\_065059012\_5408-model3.38 | -8.34306 |
| # cluster 4 - Size: 1 |  |  |
|  | XP\_065059012\_5408-model4 | -8.31668 |
| # cluster 5 - Size: 13 |  |  |
|  | XP\_065059012\_5408-model5 | -8.31128 |
|  | XP\_065059012\_5408-model5.1 | -8.31128 |
|  | XP\_065059012\_5408-model5.2 | -8.31128 |
|  | XP\_065059012\_5408-model5.3 | -8.31128 |
|  | XP\_065059012\_5408-model5.4 | -8.31128 |
|  | XP\_065059012\_5408-model5.5 | -8.31128 |
|  | XP\_065059012\_5408-model5.6 | -8.31128 |
|  | XP\_065059012\_5408-model5.7 | -8.31128 |
|  | XP\_065059012\_5408-model5.8 | -8.31128 |
|  | XP\_065059012\_5408-model5.9 | -8.31128 |
|  | XP\_065059012\_5408-model5.10 | -8.31128 |
|  | XP\_065059012\_5408-model5.11 | -8.31128 |
|  | XP\_065059012\_5408-model5.12 | -8.31128 |
|  |  |  |
